# *Lactobacillus* probiotic cell-free supernatants and vitamin D influence interleukin-6 production and mitigate oral periodontopathogens-induced cytotoxicity in FaDu cells

**DOI:** 10.3389/fmicb.2025.1578267

**Published:** 2025-04-25

**Authors:** Paola Zanetta, Veronica De Giorgis, Elettra Barberis, Marcello Manfredi, Angela Amoruso, Marco Pane, Barbara Azzimonti

**Affiliations:** ^1^Laboratory of Applied Microbiology, Department of Health Sciences (DiSS), Center for Translational Research on Allergic and Autoimmune Diseases (CAAD), School of Medicine, Università del Piemonte Orientale (UPO), Novara, Italy; ^2^Laboratory of Biological Mass Spectrometry, Department of Translational Medicine (DiMeT), Center for Translational Research on Allergic and Autoimmune Diseases (CAAD), School of Medicine, Università del Piemonte Orientale (UPO), Novara, Italy; ^3^Department of Sciences and Technological Innovation, Università del Piemonte Orientale, Alessandria, Italy; ^4^Probiotical Research S.r.l., Novara, Italy

**Keywords:** *Lactobacillus* probiotic cell-free supernatants, vitamin D, oral periodontopathogens, interleukin-6, FADU

## Abstract

Oral eubiosis is of utmost importance for local and systemic health. Consolidated habits, as excessive alcohol consumption, smoke, inappropriate oral hygiene, and western diet, exert detrimental effects on oral microbiota composition and function. This leads to caries, gingivitis, and periodontitis, also increasing the risk of preterm births, inflammation, and cancer. Thus, effective tools to contain pathobiont overgrowth and virulence and restore oral eubiosis are needed. Therefore, the effects of *Limosilactobacillus reuteri* LRE11, *Lacticaseibacillus rhamnosus* LR04, *Lacticaseibacillus casei* LC04, and their co-culture cell-free supernatants (CFSs), produced in both conventional MRS medium and a novel animal derivative-free medium named TIL, along with vitamin D, were assessed on the viability and interleukin (IL)-6 production of oral epithelial FaDu cells infected with *Aggregatibacter actinomycetemcomitans*, *Fusobacterium nucleatum*, and *Porphyromonas gingivalis*. The CFS proteomic, short chain fatty acid, and lactic acid contents were also investigated. Interestingly, probiotic CFSs and vitamin D differentially reduced the infected cell IL-6 production and counteracted the infection-induced cytotoxicity. Taken together, these results suggest that probiotics and vitamin D can reverse pathogen-induced cell damage. Since probiotic CFS effect is both strain and growth medium composition dependent, further experiments are required to deepen the probiotic and vitamin D synergic activity in this context.

## 1 Introduction

The oral cavity hosts more than 700 bacterial species, whose eubiosis is essential for oral health preservation (Le Bars et al., 2017). However, incorrect habits, as alcohol consumption, smoke, inadequate oral hygiene, and processed food, are responsible for the reduction of beneficial microorganisms, increase of pathogenic bacterial, viral, and fungal load and aggressiveness (Chattopadhyay et al., 2019; Tuominen and Rautava, 2021), and thus for oral microbiota dysbiosis (Gao et al., 2018). These conditions are key players in the onset and worsening of oral diseases, such as caries, gingivitis, periodontitis, and cancer (Gao et al., 2018). Moreover, oral dysbiosis has been also associated with extra-oral morbidities, such as infective, inflammatory, and immune-mediated conditions, like inflammatory bowel disease (Gentschew and Ferguson, 2012; Neuman and Nanau, 2012; Pickard et al., 2017; Caballero and Pamer, 2015; Li et al., 2019; Jose and Heyman, 2008; Veloso, 2011; Zhang et al., 2022; Kato et al., 2018; Liu et al., 2020; Li et al., 2020), Alzheimer (Miklossy, 1993; Riviere et al., 2002; Kamer et al., 2009; Franciotti et al., 2021; Poole et al., 2013), diabetes (Ussar et al., 2016; Cullinan et al., 2001; Emrich et al., 1991; Casarin et al., 2013), rheumatoid arthritis (McInnes and Schett, 2011; Zhang et al., 2015; Fagan, 1976; Moroi and Sato, 1975), atherosclerosis (Koren et al., 2011; Libby et al., 2002), and infective endocarditis (Sharara et al., 2016; Shelburne et al., 2014; Mitchell, 2011), and preterm birth (Hong et al., 2023; Vidmar Šimic et al., 2023; Yin et al., 2021). Due to their involvement in such conditions (Topcuoglu and Kulekci, 2015; Hajishengallis, 2015) and ability to generate volatile sulfur compounds responsible for chronic inflammation, cell proliferation, migration, invasion, tumor angiogenesis and aggressivity mainly in the oral niche, but also in distant sites (Kamarajan et al., 2020; Metsäniitty et al., 2024), the interest on the periodontopathogens *Aggregatibacter actinomycetemcomitans*, *Fusobacterium nucleatum*, and *Porphyromonas gingivalis* is growing (Chattopadhyay et al., 2019). If on one side oral eubiosis preservation is crucial to prevent the onset of oral and extra-oral pathologies or ameliorate patient’s response to therapy and life quality, on the other hand it is no more possible to count only on antibiotics due to the increase of bacterial multidrug resistances (Ardila and Vivares-Builes, 2022). In this context, probiotics may serve as effective tools to reduce the overgrowth and virulence of pathobionts, helping to restore a commensal phenotype. Recent studies demonstrate that, when used to manage oral conditions, probiotics can decrease the local abundance of pathogens and improve oral health (Seminario-Amez et al., 2017). Moreover, their effectiveness, which include that of live or dead probiotics and their metabolites, has been assessed in the prevention and treatment of several cancer types included those of the oropharyngeal niche (Legesse Bedada et al., 2020; Mohd Fuad et al., 2023; Frey-Furtado et al., 2024). Although vitamin D function is well established in bone metabolism (Feldman et al., 2014), it is now getting more interesting for its extra-skeletal positive effects, including those ones related to oral health. Severe vitamin D deficiency has been associated with rachitic tooth and caries (Botelho et al., 2020), periodontitis (Najeeb et al., 2016; Jagelavičienė et al., 2018; Garcia et al., 2011; Grant and Boucher, 2010; Stein et al., 2014), and oral neoplastic lesions (Fathi et al., 2019). Furthermore, a growing body of literature is underlying vitamin D role in the maintenance of a healthy gut microbiota, being altered when deficient or when its receptor is depleted (Ooi et al., 2013; Cantorna et al., 2014; Jin et al., 2015; Wu et al., 2015). In turn, specific probiotic supplementation has been associated to both vitamin D and vitamin D receptor (VDR) increase (Wu et al., 2015; Raveschot et al., 2020). In addition, the co-supplementation of vitamin D and probiotics has shown beneficial effects in the polycystic ovary syndrome (Ostadmohammadi et al., 2019) and obesity (Karbaschian et al., 2018), being also a possible tool for improving inflammatory bowel disease patient’s status, as demonstrated in the disease mouse model (Jagelavičienė et al., 2018). For these reasons we hypothesized that, due to the potential beneficial role of probiotics and vitamin D on gut microbiota, a similar synergistic effect could be also exploited on oral microbiota. Thus, we investigated the effect of *Limosilactobacillus reuteri* LRE11 (DSM 33827), *Lacticaseibacillus rhamnosus* LR04 (DSM 16605), *Lacticaseibacillus casei* LC04 (DSM 33400), and their co-culture (L3) cell-free supernatants (CFSs) and/or that of vitamin D in reducing cell cytotoxicity and interleukin (IL)-6 production by a human hypopharyngeal squamous carcinoma (FaDu) cell line after co-infection with *A. actinomycetemcomitans, F. nucleatum*, and *P. gingivalis*. This cellular model, equipped with key immune receptors, is widely used in *in vitro* study inflammation of the oral and pharyngeal regions. As already published by our group, since the probiotic effect also depends on culture conditions (Zanetta et al., 2023), the CFSs were produced both in the standard MRS and in the novel animal derivative-free TIL media. In addition, the L3 CFS was also tested after enzymatic digestion with proteinase K (PK) and trypsin (TRY). Finally, a proteomic characterization of the probiotic CFSs was done, together with the determination of lactic acid and short chain fatty acids (SCFAs) production. Interestingly, probiotic CFSs and vitamin D significantly reduced IL-6 production and cell cytotoxicity upon infection with the three periodontopathogens. Since the effect observed was strain specific, further experiments are needed to clarify the molecular mechanisms underlying the interaction among probiotics and vitamin D in this context.

## 2 Results

### 2.1 Probiotic CFS and vitamin D effect on FaDu cell line

To exclude that the CFSs produced by the probiotic strains in their culture media could *per se* induce any cytotoxicity on oral FaDu cells and determine the most appropriate probiotic CFS concentration before their *in vitro* biological activity evaluation, a preliminary cell viability assay was conducted at 4 h of incubation.

**Figure 1:**
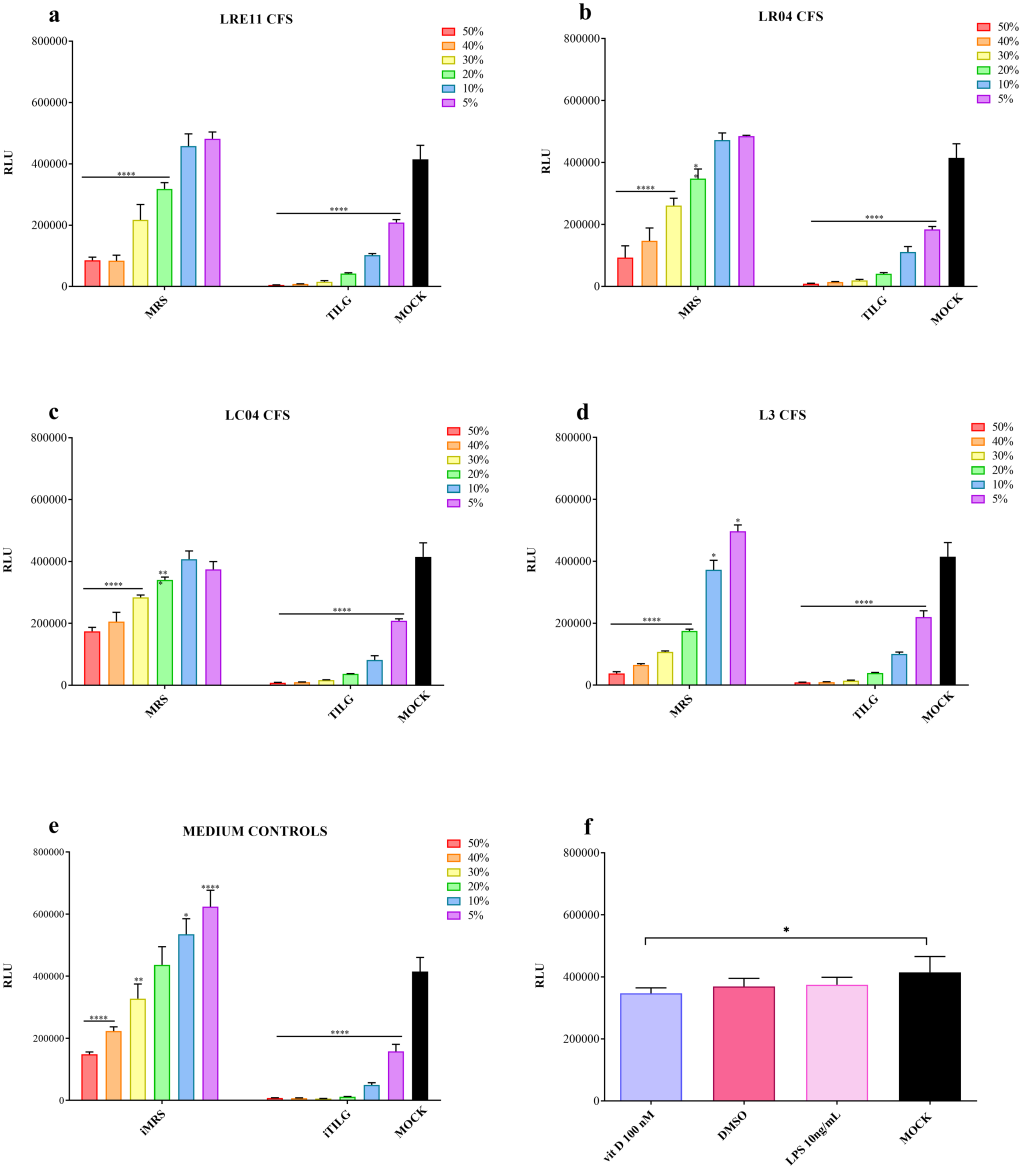
Probiotic CFS, vitamin D, and LPS effect on FaDu cells at 4 h. A cell viability assay was conducted after 4 h of treatment with different concentrations of **(a)** LRE11, **(b)** LR04, **(c)** LC04, **(d)** L3 CFSs, **(e)** iMRS/iTILG, and with **(f)** vitamin D at 100 nM, DMSO, and LPS at 10 ng/mL. All data are represented as the mean of three independent experiments ± SD. *p < 0.05; **p < 0.01; ***p < 0.001; ****p < 0.0001. Differences on the graph are compared to the untreated control (MOCK). CFS, cell-free supernatant; RLU, relative luminescent unit.

**Figure 2:**
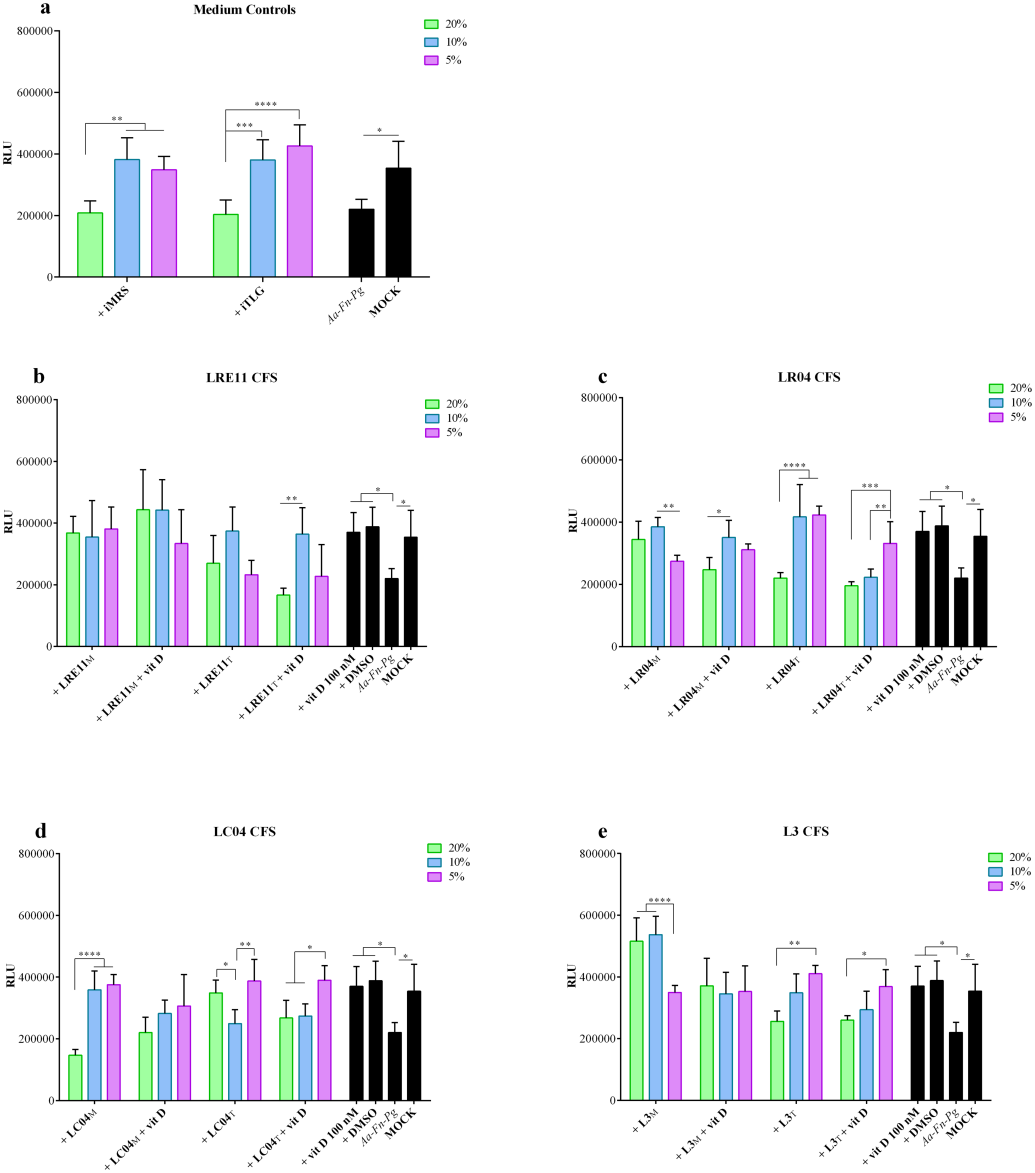
Probiotic CFS and vitamin D effect on infected FaDu cell line viability. Viability assay was conducted 4 h post treatment with different concentrations of **(a)** iMRS/iTILG, **(b)** LRE11, **(c)** LC04, **(d)** LR04, and **(e)** L3 CFSs also in combination with vitamin D at 100 nM. All data are represented as the mean of three independent experiments ± SD. *p < 0.05; **p < 0.01; ***p < 0.001; ****p < 0.0001. CFS, cell-free supernatant; RLU, relative luminescent unit.

For the MRS-produced CFSs, a significant reduction in viability was noted at the 50 and 40% concentrations (*p* < 0.0001; Figures 1a–d). The 30% concentration also exhibited significant toxicity for all CFSs compared to the untreated control (mock; *p* < 0.0001; Figures 1a–d). Moreover, the 20% concentration significantly reduced FaDu cell viability compared to the mock, as shown in Figures 1a–d. The 10 and 5% CFS concentrations did not show any significant difference from the mock, except for L3 CFS (*p* < 0.05; Figure 1d). For the TIL-produced CFSs, they similarly and significantly reduced FaDu cell viability (*p* < 0.0001; Figures 1a–d). The iTILG control showed a higher toxicity when compared to the iMRS one (Figure 1e), while only vitamin D alone significantly reduced FaDu cell viability compared to the mock (*p* < 0.05; Figure 2f). Despite these results, the 20% concentration was maintained for both the viability assay on infected cells and the IL-6 ELISA experiments, as the presence of bacterial pathogens capable of metabolizing certain CFS components could still yield beneficial effects in preventing infection outcomes.

Vitamin D 100 nM concentration, which corresponds to 38.4 ng/mL, was selected based on literature data and considering that the physiological blood range value should be between 30 and 100 ng/mL, as established by different scientific societies and countries (Patria et al., 2019). Considering the need to use vitamin D in combination with probiotic CFSs and its liposolubility in DMSO, a viability assay was conducted also with this compound as control. The same was done with LPS, at a concentration of 10 ng/mL since it was used as control in the ELISA experiments.

### 2.2 Probiotic CFS effect on infected FaDu cell line

The viability of FaDu cells 4 h post infection with a mixture of *A. actinomycetemcomitans*, *F. nucleatum*, and *P. gingivalis* at 100 MOI, and with 20, 10, and 5% probiotic CFS concentrations combined with vitamin D is shown in Figure 2. The iMRS and iTILG controls were used with the same v/v ratios applied to treat the infected cells.

Evaluating cell viability at 4 h provided reliable results, preventing alterations from uncontrolled pathogen growth and medium acidification over time. Additionally, oral pathogens, such as *A. actinomycetemcomitans, F. nucleatum,* and *P. gingivalis*, can adhere to epithelial cells within minutes to a few hours (Li et al., 2015). Their adhesion and invasiveness are also significantly enhanced when incubated together rather than individually (Mu et al., 2020).

The combination of the three pathogens slightly reduced FaDu cell viability compared to the mock control (*p* < 0.05, Figure 2). The prokaryotic media significantly improved cell viability at 10% (iMRS and iTILG vs. infected cells, *p* < 0.05; Figure 2a) and 5% (iMRS vs. infected cells, *p* < 0.05, iTILG, *p* < 0.01; Figure 2a), without significant differences compared to the mock. At 20% v/v, neither medium affected infected FaDu cell viability (Figure 2a). When vitamin D and DMSO were used, cell viability was restored to mock control levels (*p* < 0.05 vs. infected cells; not significant vs. mock; Figures 2b–e). However, since no significant differences were observed between vitamin D and DMSO, the effect cannot be attributed to vitamin D alone.

Among LRE11 CFS concentrations, there were no significant differences, except between the TIL-produced CFS at 20% + vitamin D, and 10% + vitamin D (Figure 2b). All concentrations of the MRS-produced LRE11 CFS significantly reduced infection-induced cytotoxicity (*p* < 0.05 for 20 and 10%, *p* < 0.01 for 5%; Figure 2b), restoring cell viability to mock levels. A similar effect was observed when vitamin D was added, but only at 20 and 10% concentrations (*p* < 0.01 vs. infected control; Figure 2b). However, no significant differences were observed between CFS treatment alone and CFS + vitamin D, indicating that vitamin D did not enhance CFS efficacy. For the probiotic media, only the 20% v/v ratio significantly differed from the iMRS 20% control (*p* < 0.05 for CFS alone; *p* < 0.001 for CFS + vitamin D), making it the only concentration able to significantly reduce cytotoxicity despite the MRS impact on infection. When TIL-produced LRE11 CFS was used, only the 10% v/v ratio significantly restored viability compared to infected FaDu cells (*p* < 0.05; Figure 2b), whether used alone or with vitamin D. However, no significant differences were found between these conditions and the iTILG control at 10%, indicating that the observed effect could not be attributed to the CFS alone.

Figure 2c presents the graph for LR04 CFS at various concentrations. The MRS-produced CFS significantly reduced cell toxicity compared to the infected control at both 20 and 10% concentrations (*p* < 0.05 and *p* < 0.01, respectively; Figure 2c). When vitamin D was added, only the 10% LR04 CFS maintained a significant difference compared to infected cells (*p* < 0.05; Figure 2a), though there was no significant difference between this condition and the one without vitamin D. For the iMRS controls, only the 20% LR04 CFS showed a significant difference compared to the 20% iMRS (*p* < 0.001), indicating a specific protective activity of the CFS. In this case, adding vitamin D significantly reduced the activity of 20% LR04 CFS (*p* < 0.01; Figure 2c). In the TIL-produced CFS treatments, both 20 and 10% LR04 CFS significantly reduced infected FaDu cell toxicity (*p* < 0.01 and *p* < 0.001, respectively; Figure 2c), though no significant difference was observed compared to the respective iTILG controls. Furthermore, the addition of vitamin D significantly worsened the effects of 10 and 5% LR04 CFS (*p* < 0.0001 and *p* < 0.001, compared to the same conditions without vitamin D; Figure 2c).

LC04 CFS significantly reduced infected cell toxicity under the following conditions: 5% LC04 CFS in MRS + vitamin D (*p* < 0.01; Figure 2d), 5% LC04 CFS in TIL (*p* < 0.05; Figure 2d), and 5% LC04 CFS in TIL + vitamin D (*p* < 0.01; Figure 2d). However, no significant differences were observed when comparing these conditions with the respective medium controls. Figure 2d also shows the differences between the various CFS concentrations for the same condition.

Interestingly, when the three probiotics were co-cultured, their L3 CFS in MRS significantly decreased cytotoxicity compared to the untreated infected cells (*p* < 0.0001 for 20 and 10% L3 CFS, *p* < 0.05 for 5% L3 CFS, and 20, 10, and 5% L3 CFS + vitamin D; Figure 2e). L3 CFS at 20 and 10% also showed a significant difference compared to the respective iMRS controls (*p* < 0.0001 and *p* < 0.01, respectively; Figure 2e). However, vitamin D significantly reduced the activity of CFS (*p* < 0.01; Figure 2e). For the TIL-produced L3 CFS, only the 10 and 5% v/v ratios demonstrated an effect in preventing cell death (*p* < 0.05 and *p* < 0.01 when compared to infected cells, respectively; Figure 2e). When vitamin D was added, only 5% L3 CFS maintained a significant activity (*p* < 0.05; Figure 2e), although none of these conditions showed a significant difference compared to the corresponding iTILG control.

### 2.3 Probiotic CFS effect on FaDu cell line IL-6 production

**Figure 3:**
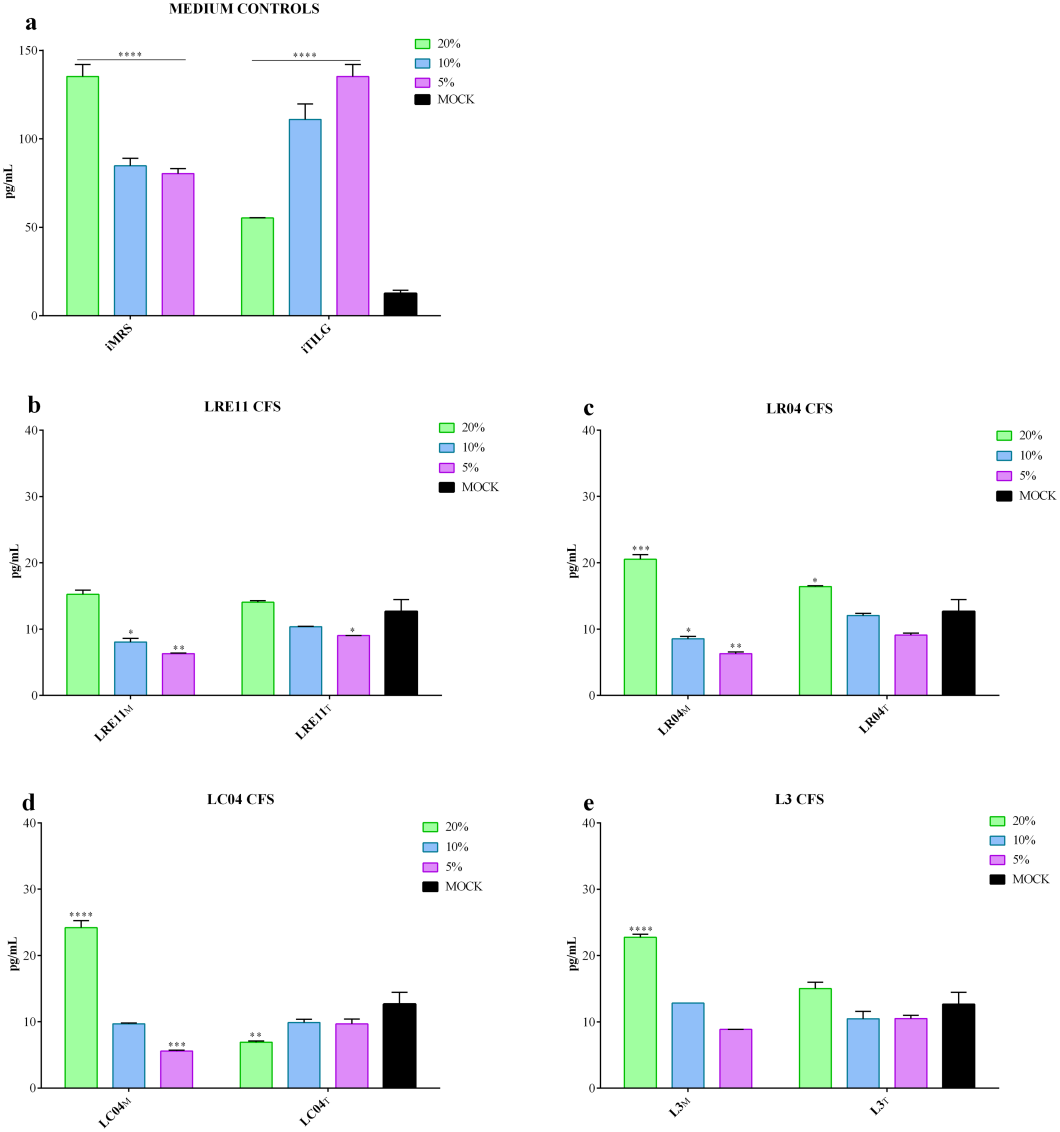
Probiotic CFS effect on IL-6 production by FaDu cells 4 h post treatment. IL-6 production was evaluated after 4 h of treatment with different concentrations of **(a)** iMRS/iTILG, **(b)** LRE11, **(c)** LR04, **(d)** LC04, and **(e)** L3 CFSs. All data are represented as the mean of three independent experiments ± SD. *p < 0.05; **p < 0.01; ***p < 0.001; ****p < 0.0001. Differences on the graph are compared to the untreated control (MOCK). CFS, cell-free supernatant; RLU, relative luminescent unit.

When iMRS and iTILG were used as control media for this experiment, they significantly increased IL-6 production by FaDu cells after 4 h of treatment (*p* < 0.0001; Figure 3a). Consequently, data were adjusted and normalized on the effect of the medium alone, aiming to isolate the contribution of CFS by-products. MRS-produced LRE11 CFS demonstrated to reduce IL-6 production by tumoral cells at 10 and 5% concentrations, as shown by the statistical analysis presented in the graph (Figure 3b). A similar effect was observed for 5% LRE11 CFS in TIL. LR04 CFS exhibited a similar pattern, except that both MRS-and TIL-produced CFS at 20% significantly increased IL-6 production, as shown in Figure 3c. Additionally, 20% LC04 and L3 CFSs in MRS also induced IL-6 production, while 5% LC04 in MRS and 20% LC04 in TIL reduced it (Figures 3d,e). The MRS-produced CFSs generally performed a better activity compared to the TIL-produced ones, with some exceptions for LR04 and LC04.

### 2.4 Probiotic CFS effect on infected FaDu cell line IL-6 production

**Figure 4:**
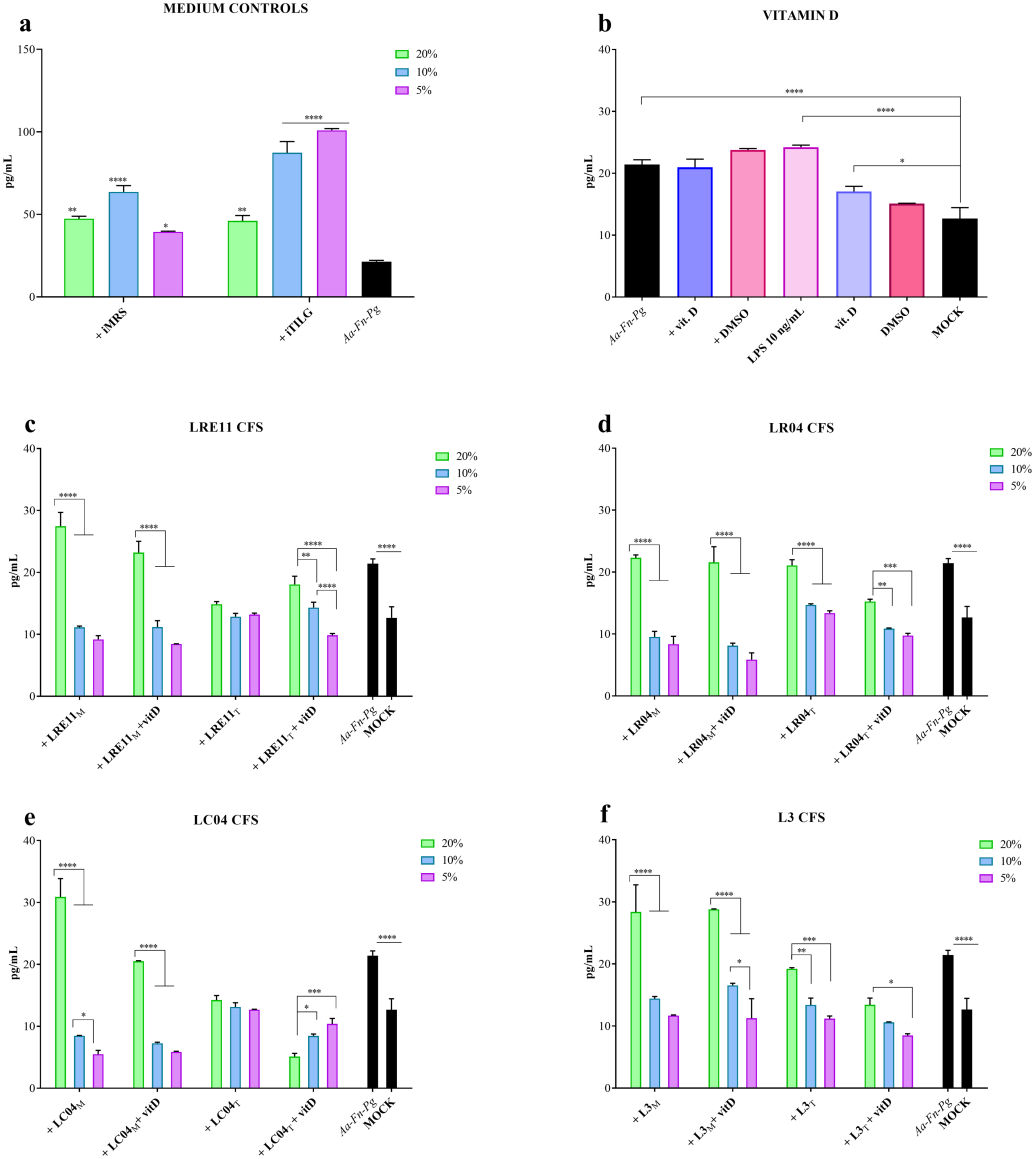
Probiotic CFS effect, with or without vitamin D, on IL-6 production by infected FaDu cells 4 h post treatment. IL-6 production by infected FaDu cells was evaluated after 4 h of treatment with different concentrations of **(a)** iMRS/iTILG, with **(b)** vitamin D (100 nM) and LPS (10 ng/mL), **(c)** LRE11, **(d)** LR04, **(e)** LC04, and **(f)** L3 CFSs. All data are represented as the mean of three independent experiments ± SD. *p < 0.05; **p < 0.01; ***p < 0.001; ****p < 0.0001; differences on the graph are compared to the untreated control (MOCK). CFS, cell-free supernatant; RLU, relative luminescent unit.

When FaDu cells were infected with *A. actinomycetemcomitans*, *F. nucleatum*, and *P. gingivalis*, the iMRS and iTILG media controls still significantly increased IL-6 production in infected cells, creating a confounding effect (Figure 4a). To address this, data were normalized based on the medium controls according to the concentration used, aiming to eliminate the medium confounding effects.

Figure 4b shows the individual effect of vitamin D, with DMSO as a control, on both infected and non-infected cells. The co-infection significantly increased IL-6 production compared to the mock control, and this increase was not affected by either vitamin D or DMSO (Figure 4b). Vitamin D and DMSO alone slightly increased IL-6 production at 4 h (Figure 4b). LPS at 10 ng/mL, used as a positive control for inflammation induction, resulted in a similar outcome to the pathogen infection (Figure 4b).

**Figure 5:**
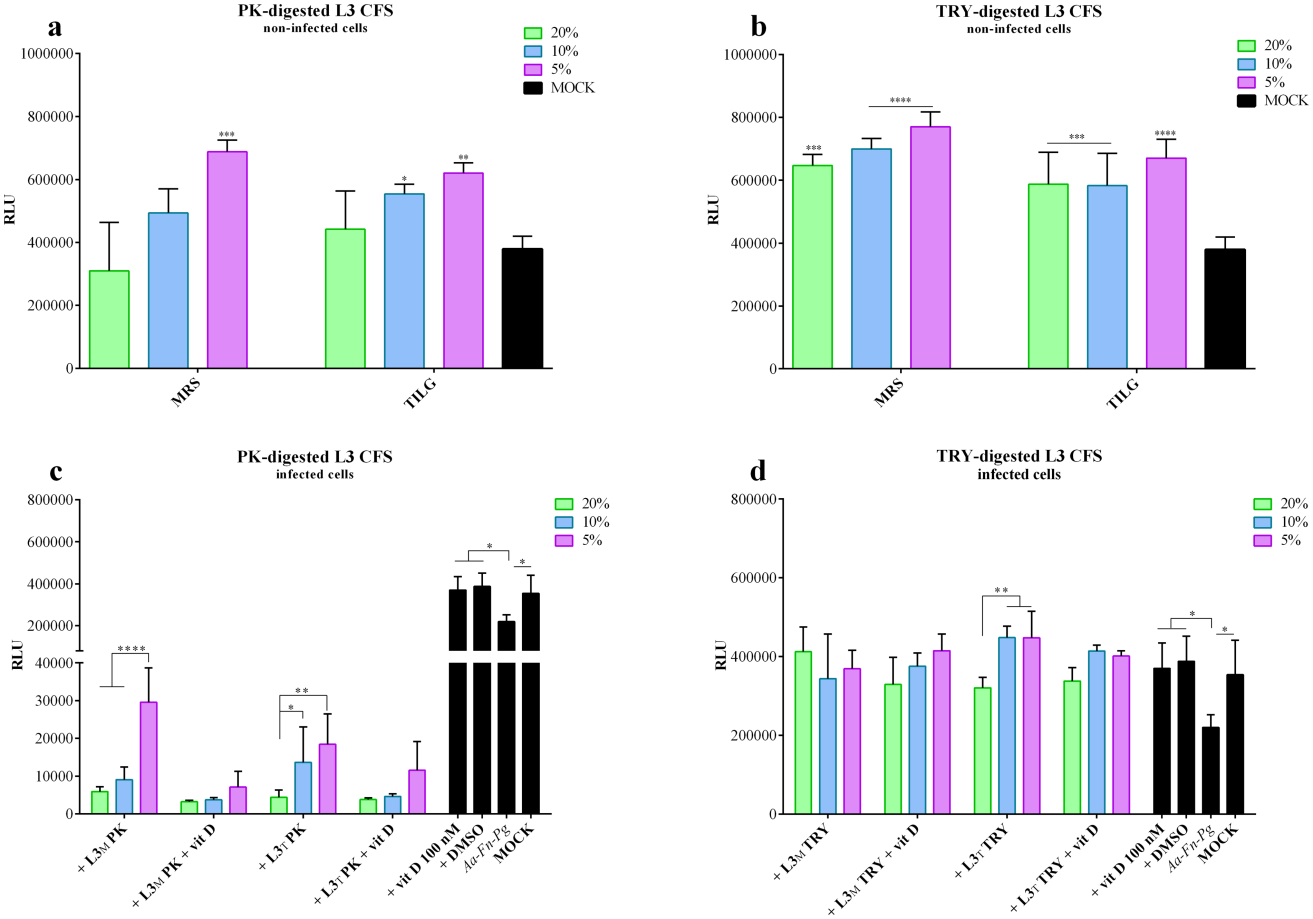
PK-or TRY-digested L3 CFS and vitamin D improvement of infected cell viability. Viability assay was conducted on non-infected FaDu cells after 4 h of treatment with different concentrations of **(a)** PK-digested L3 CFS, and **(b)** TRY-digested L3 CFS; or on infected cells after 4 h of treatment with different concentrations of **(c)** PK-digested L3 CFS, and **(d)** TRY-digested L3 CFS also in combination with vitamin D at 100 nM. All data are represented as the mean of three independent experiments ± SD. *p < 0.05; **p < 0.01; ***p < 0.001; ****p < 0.0001. CFS, cell-free supernatant; RLU, relative luminescent unit.

LRE11 CFS at 20% produced in MRS significantly increased IL-6 production compared to both infected cells (*p* < 0.05; Figure 4c) and the mock (*p* < 0.0001; Figure 4c). Instead, the 10 and 5% concentrations significantly reduced IL-6 production compared to the infected cells (*p* < 0.0001; Figure 4c). When cells were co-treated with vitamin D, a similar pattern was observed, with no significant effect at 20%, but a significant reduction at 10 and 5% concentrations (*p* < 0.0001; Figure 4c). No significant differences were seen between LRE11 CFS alone and LRE11 CFS + vitamin D, except at 20%, where the addition of vitamin D significantly reduced IL-6 levels compared to CFS alone (*p* < 0.01; Figure 4c). All the concentrations of TIL-produced LRE11 CFS significantly reduced IL-6 levels compared to the infected control, restoring IL-6 levels to the one of the mock (*p* < 0.01 for 20%, *p* < 0.001 for 10 and 5%; Figure 4c). When vitamin D was added, the effect was less pronounced, with IL-6 levels at 20% significantly increasing compared to both LRE11 CFS alone (*p* < 0.05) and the mock (*p* < 0.01; Figure 4c). LRE11 CFS at 10% + vitamin D had the same outcome than the CFS alone significantly decreased IL-6 production upon infection (*p* < 0.01; Figure 5c), while the co-treatment with vitamin D and 5% LRE11 CFS showed an IL-6 reduction upon infection (*p* < 0.0001) and also compared to the CFS treatment alone (*p* < 0.05; Figure 5c). Comparing the two media, the TIL-produced 20% CFS alone resulted in a greater IL-6 reduction than the MRS-produced CFS (*p* < 0.0001), including in the presence of vitamin D (*p* < 0.01; Figure 4c). At 5%, MRS-produced LRE11 CFS was more effective (*p* < 0.05), while at 10%, MRS-LRE11 CFS showed slightly higher activity than TIL-LRE11 CFS when vitamin D was added (*p* < 0.05; Figure 4c).

For LR04 CFS, all the 20% concentrations did not reduce IL-6 production upon infection, except for the TIL-produced CFS + vitamin D (*p* < 0.01 vs. infection control; Figure 4d), which was more effective than TIL-CFS alone (*p* < 0.0001) and better than the same condition in MRS (*p* < 0.001). All 10 and 5% concentrations significantly decreased IL-6 production compared to the infected control (*p* < 0.0001 for most, except *p* < 0.01 for TIL-LR04 CFS at 10%, and *p* < 0.001 for TIL-LR04 CFS at 5%; Figure 4d). Interestingly, MRS-LR04 CFS at 5%, with and without vitamin D, and TIL-LR04 CFS + vitamin D reduced IL-6 levels when compared to the mock (*p* < 0.05, *p* < 0.001, and *p* < 0.0001, respectively; Figure 4d). At 10%, the MRS-CFS was more effective than the TIL-CFS (*p* < 0.001), achieving a comparable effect only with vitamin D addition (*p* < 0.01 for TIL-CFS 10% with and without vitamin D; Figure 5d). At 5%, MRS-CFSs with and without vitamin D performed better than the relative conditions in TIL (*p* < 0.01), but vitamin D improved TIL-LR04 CFS efficacy at this concentration compared to the treatment without vitamin D (*p* < 0.05; Figure 4d).

MRS-produced LC04 CFS at 20% slightly counteracted infection-induced inflammation but still significantly increased IL-6 levels compared to the infection control (*p* < 0.0001; Figure 4e), while vitamin D significantly reduced this effect (*p* < 0.0001), bringing IL-6 levels to those seen during infection. Conversely, 20% TIL-CFS significantly reduced IL-6 production upon infection (*p* < 0.01) and, when vitamin D was added, IL-6 levels were lower than both the infected control and the mock (*p* < 0.0001; Figure 4e). In this condition, the TIL-CFS proved to be significantly better than the MRS-produced one (*p* < 0.0001 for all 20% conditions). Conversely, at lower concentrations (10 and 5%), the MRS-CFS was more effective than TIL-CFS (*p* < 0.01 between LC04 CFS + vitamin D at 10 and 5%, and *p* < 0.0001 between LC04 CFS at 5%). However, for both LC04 CFSs, it could be observed that at 10% there was a significant reduction in IL-6 production compared to the infected cells (*p* < 0.0001 for MRS-LC04 with and without vitamin D, and TIL-LC04 CFS + vitamin D; *p* < 0.001 for TIL-LC04 CFS without vitamin D; Figure 4e). Moreover, vitamin D further reduced IL-6 when compared to the mock (*p* < 0.01 for MRS-CFS, *p* < 0.05 for TIL-CFS; Figure 4e). Both MRS-LC04 CFS at 5%, with or without vitamin D significantly reduced IL-6 production compared either to the infected cells (*p* < 0.0001) and the mock (*p* < 0.001; Figure 4e), without any difference between treatments. TIL-produced CFS at 5% effectively reduced IL-6 after infection (*p* < 0.001), with a greater effect when vitamin D was added (*p* < 0.0001), which reduced IL-6 levels even when compared to the mock (*p* < 0.0001).

When L3 CFS was used, it was observed that the MRS-produced one at 20% v/v ration, both with and without vitamin D, did not decrease IL-6 production upon FaDu infection, but it significantly increased it compared to the infection control (*p* < 0.05; Figure 4f). In contrast, 20% TIL-L3 CFS + vitamin D significantly reduced infection-induced IL-6 production (*p* < 0.05; Figure 4f), with vitamin D significantly improving CFS efficacy (*p* < 0.05). At lower concentrations, MRS-L3 CFS significantly inhibited IL-6 increase after infection (*p* < 0.05 for MRS-L3 CFS 10%, and *p* < 0.01 for 5%, compared to the infection control; Figure 4f). When vitamin D was added, only the 5% concentration resulted in a significant difference when compared to the infected cells (*p* < 0.01; Figure 4f), but without significantly improving the effect of the CFS alone. Similar results were seen for TIL-L3 CFS (*p* < 0.05 for 10%, *p* < 0.01 for 5% vs. the infected control; Figure 4f), with vitamin D significantly increasing the 5% L3 CFS ability in reducing IL-6 levels upon infection (*p* < 0.0001; Figure 4f).

Interestingly, vitamin D improved the anti-inflammatory activity of certain probiotic CFSs, including 20% MRS-LRE11; 20, 10, and 5% TIL-LR04; 5% MRS-LR04; 20, 10, and 5% TIL-LC04; and 5% TIL-L3. Moreover, some of the treatments not only reduced IL-6 levels induced by FaDu pathogen infection, but also significantly lowered IL-6 levels compared to the baseline observed in the mock, as it was seen with 10 and 5% MRS-LR04 with and without vitamin D, 10 and 5% MRS-LC04 with and without vitamin D, and 20% TIL-LC04 + vitamin D.

### 2.5 Effects of L3 CFS digested with proteinase K (PK) or trypsin (TRY) on infected FaDu cell line viability and IL-6 production

L3 CFS was PK and TRY digested to identify the component categories which might be responsible for the effects described above. This kind of digestion inactivates all the protein components present in the CFS, giving the possibility to determine whether the active substances belong to this biochemical group. Specifically, if the active molecules are proteins, the effect will be reduced or lost upon digestion; vice versa, the observed outcome may remain unchanged or improve.

Figure 5 represents the cell viability toxicity compared to the non-digested ones. In particular, 20% L3 CFS, when compared to the digested versions, induced significantly lower viability of FaDu cells (*p* < 0.0001) in both MRS and TIL. At the same concentration, TRY-digested L3 CFS exhibited higher viability than PK-digested CFS (*p* < 0.05). At 10%, MRS-L3 CFS caused significantly lower viability compared to the TRY-digested one (*p* < 0.0001), while in TIL it significantly reduced viability with respect to both PK and TRY-digested CFSs (*p* < 0.0001). Additionally, the 10% TRY-treated L3 CFS in MRS was less toxic than the PK-digested version (*p* < 0.001). When the 5% concentration was used, the only significant difference was that untreated L3 CFS exhibited higher toxicity than both the enzyme-treated ones (*p* < 0.01 vs. PK-digested in MRS, *p* < 0.0001 vs. TRY-digested in MRS and TIL, and PK-digested in TIL).

In infected cells, the PK-digested L3 CFS in MRS at all concentrations, with or without vitamin D, significantly reduced cell viability when compared to the infected control (*p* < 0.001 for all; Figure 5c) and to the mock (*p* < 0.0001 for all; Figure 5c). Moreover, the differences were also significant when compared to the respective iMRS and iTILG controls (*p* < 0.0001). At 5%, the PK-digested L3 CFS in TIL was less effective than in MRS (*p* < 0.01; Figure 5c), and the addition of vitamin D further decreased its efficacy (*p* < 0.0001 for PK L3 CFS 5% vs. PK L3 CFS + vitamin D in MRS; *p* < 0.05 at 10%; Figure 5c).

With TRY-digested L3 CFS, instead, an opposite outcome was observed (Figure 5d). The MRS-produced TRY-digested CFS significantly improved infected cell viability at 20 and 5% (*p* < 0.01; Figure 5d), while at 10 and 5% when vitamin D was added (*p* < 0.01; Figure 5d), though the addition of vitamin D did not alter the CFS effect. When TIL-produced L3 CFS was used, a significant improvement in infected cell viability was observed at 10 and 5% concentrations (*p* < 0.001), and at 20, 10, and 5% when vitamin D was added (*p* < 0.05, *p* < 0.001, *p* < 0.01, results at 4 h on non-and infected cells. The digested L3 CFSs in both media did not show any significant toxic effect on FaDu cells; instead, they increased cell viability at certain concentrations when compared to the mock control, as shown by the statistic reported in Figures 5a,b. For the PK-digested L3 CFS, only the 5% concentration in MRS and the 10 and 5% concentrations in TIL demonstrated this effect, while all treatments of the TRY-digested L3 CFS did (Figures 5a,b). The digested L3 CFSs showed significantly lower respectively; Figure 5d). However, vitamin D did not enhance or reduce the CFS efficacy also in this case. No statistically significant differences were observed between digested and non-digested L3 CFS. However, at 20%, PK-digested L3 CFS had the lowest efficacy in preventing infected cell death compared to both undigested and TRY-digested L3 CFS, in both MRS and TIL, with or without vitamin D (*p* < 0.0001). At 10%, a similar difference was observed, including a significant decrease in infected cell viability with TRY-digested L3 CFS in MRS compared to the undigested one (*p* < 0.0001), and an increase with TRY-treated L3 CFS in TIL + vitamin D when compared to the undigested one (*p* < 0.05). At 5%, PK-digested L3 CFS was significantly less effective than TRY-digested and undigested versions, regardless of the medium used or vitamin D supplementation (*p* < 0.0001 for all conditions).

**Figure 6:**
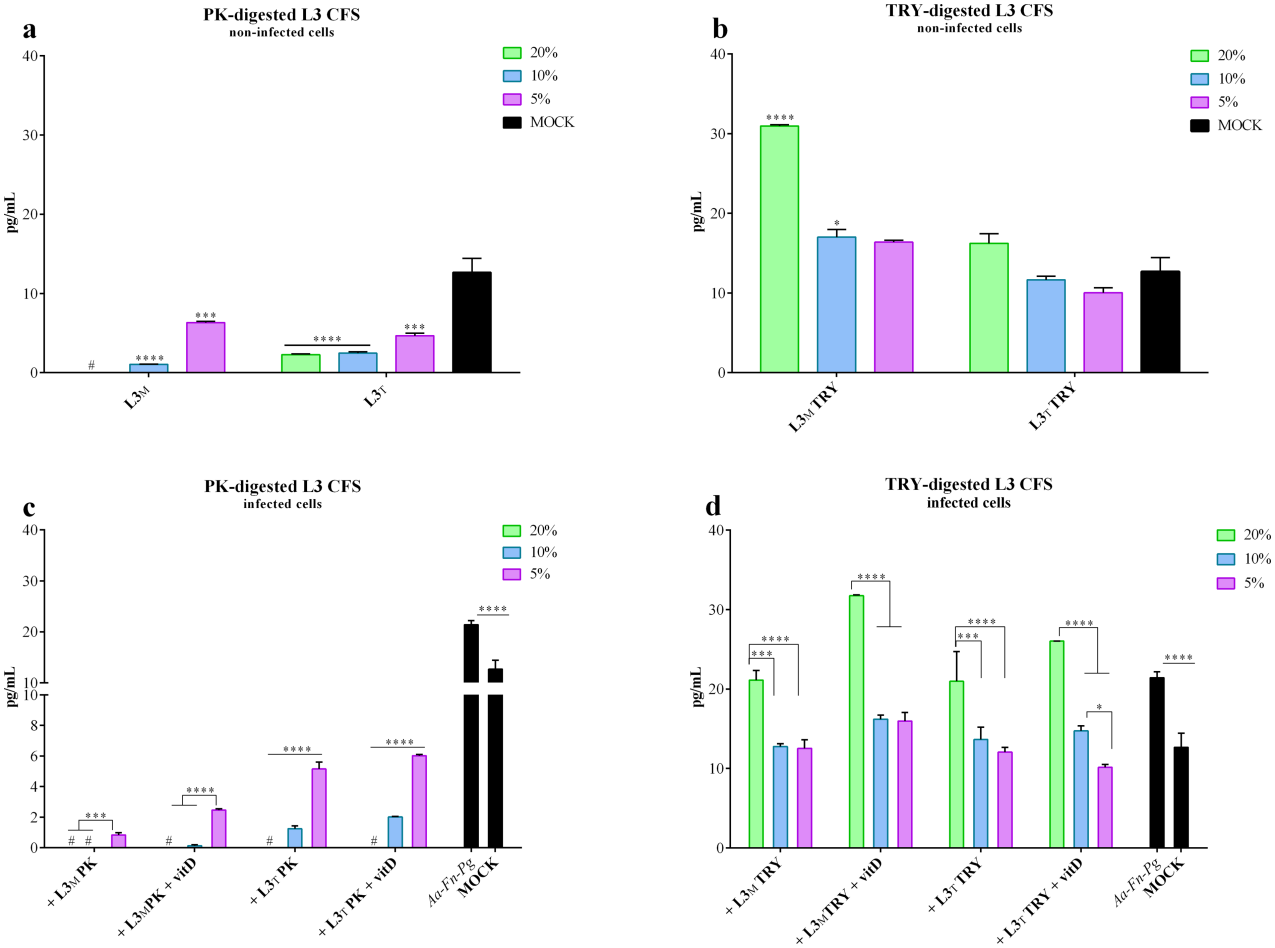
PK-or TRY-digested L3 CFS and vitamin D and IL-6 production reduction by infected cells. IL-6 production was evaluated on non-infected FaDu cells after 4 h treatment with different concentrations of **(a)** PK-digested L3 CFS, and **(b)** TRY-digested L3 CFS; or on infected FaDu cells after 4 h treatment with different concentrations of **(c)** PK-digested L3 CFS, and **(d)** TRY-digested L3 CFS also in combination with vitamin D at 100 nM. All data are represented as the mean of three independent experiments ± SD. *p < 0.05; **p < 0.01; ***p < 0.001; ****p < 0.0001. #, the measurements were all below the detection limit; CFS, cell-free supernatant; RLU, relative luminescent unit.

The evaluation of IL-6 production following treatment with digested L3 CFS is shown in Figures 6a,b. Interestingly, PK-digested L3 CFS significantly reduced IL-6 production compared to the mock control at all tested concentrations and when produced in both MRS and TIL media, as illustrated in Figure 6a. For the 20% concentration of the MRS-produced CFS, IL-6 levels were even below the detection limit. In contrast, TRY-digested L3 CFS increased basal IL-6 production in FaDu cells, which was significant when compared to the mock for the 20 and 10% MRS-produced CFS (*p* < 0.0001 and *p* < 0.05, respectively; Figure 6b).

In all the conditions tested, PK-digested L3 CFS effectively reduced IL-6 production, not only compared to the infected FaDu cells (*p* < 0.0001) but also compared to the mock (*p* < 0.0001; Figure 6c). The TIL CFS was less effective than the MRS-produced one upon digestion (*p* < 0.0001 for both 10 and 5% concentrations in MRS and TIL, with and without vitamin D; Figure 6c). Among the different L3 CFSs, at 20% a significant increase in IL-6 production was observed for the TRY-treated CFS when compared to the untreated and PK-digested ones. PK-treated CFS also significantly reduced IL-6 compared to both the untreated CFS in MRS and TIL (*p* < 0.0001) and TRY-digested CFS in MRS (*p* < 0.0001). At 10%, PK-digested CFS still showed significantly lower IL-6 levels than the untreated and TRY-digested CFSs in both MRS and TIL, while the untreated CFS in MRS was more effective than the TRY-treated one (*p* < 0.0001). The same pattern and significance were observed for the 5% concentration.

For the TRY-digested L3 CFS, the 20% concentration did not effectively reduce IL-6 production upon infection regardless of vitamin D presence. At 10 and 5%, the digested CFS in MRS significantly countered the IL-6 increase upon infection (*p* < 0.01; Figure 6d), and the addition of vitamin D did not improve this outcome. Results were similar for the TIL-produced digested CFS (*p* < 0.01 for 10% concentration, *p* < 0.0001 for 5% alone and + vitamin D, *p* < 0.05 for 5% alone; Figure 6d), with TRY-digested L3 CFS 5% in TIL + vitamin D being more effective than the same condition in MRS (*p* < 0.01). Among all L3 CFSs tested, PK-digested CFS at 20% was the most effective in reducing IL-6 levels compared to TRY-treated and untreated CFSs, regardless of the medium or vitamin D presence (*p* < 0.0001). Comparing untreated L3 CFS with the TRY-treated one, the former was better in reducing IL-6 production in MRS without vitamin D (*p* < 0.0001), while the latter was more effective in the presence of vitamin D (*p* < 0.0001). The same results were observed at the 10% concentration when comparing PK-treated, untreated, and TRY-treated L3 CFSs. At 5%, PK-digested L3 CFS again showed significantly lower IL-6 levels compared to the untreated and TRY-treated ones in MRS with and without vitamin D, and in TIL without vitamin D (*p* < 0.0001 for MRS, *p* < 0.001 for TIL). Moreover, MRS L3 CFS + vitamin D was more effective in reducing IL-6 levels than TRY-treated L3 CFS in MRS + vitamin D (*p* < 0.05).

### 2.6 Probiotic CFS preliminary characterization

Results from a preliminary L3 CFS characterization are listed in Table 1. Firstly, probiotic culture pH, protein and lactic acid content of the CFSs produced in both MRS and TIL were measured. Although the protein concentration increased only for the L3 CFS produced in TIL when compared to the MRS one, for all the other CFSs a decrease in the pH value was observed in TIL, followed by a significant increase in lactic acid production in this medium with respect to the MRS-produced CFSs (*p* < 0.0001).

**Table 1:** *Lactobacillus* CFS preliminary characterization.

	MRS-produced CFS			TIL-produced CFS		
Probiotic Strain	pH	Protein concentration (mg/mL)	Lactic acid concentration (g/L)	pH	Protein concentration (mg/mL)	Lactic acid concentration (g/L)
LRE11	4.38	9.25	4.85	3.8	10.84	12.25
LR04	4.24	10.79	8.86	3.8	11.41	15.51
LC04	3.99	10.27	14.03	3.8	10.91	15.13
L3	4.25	10.38	9.37	3.8	14.31	13.32

### 2.7 Probiotic CFS proteomic analysis

**Figure 7:**
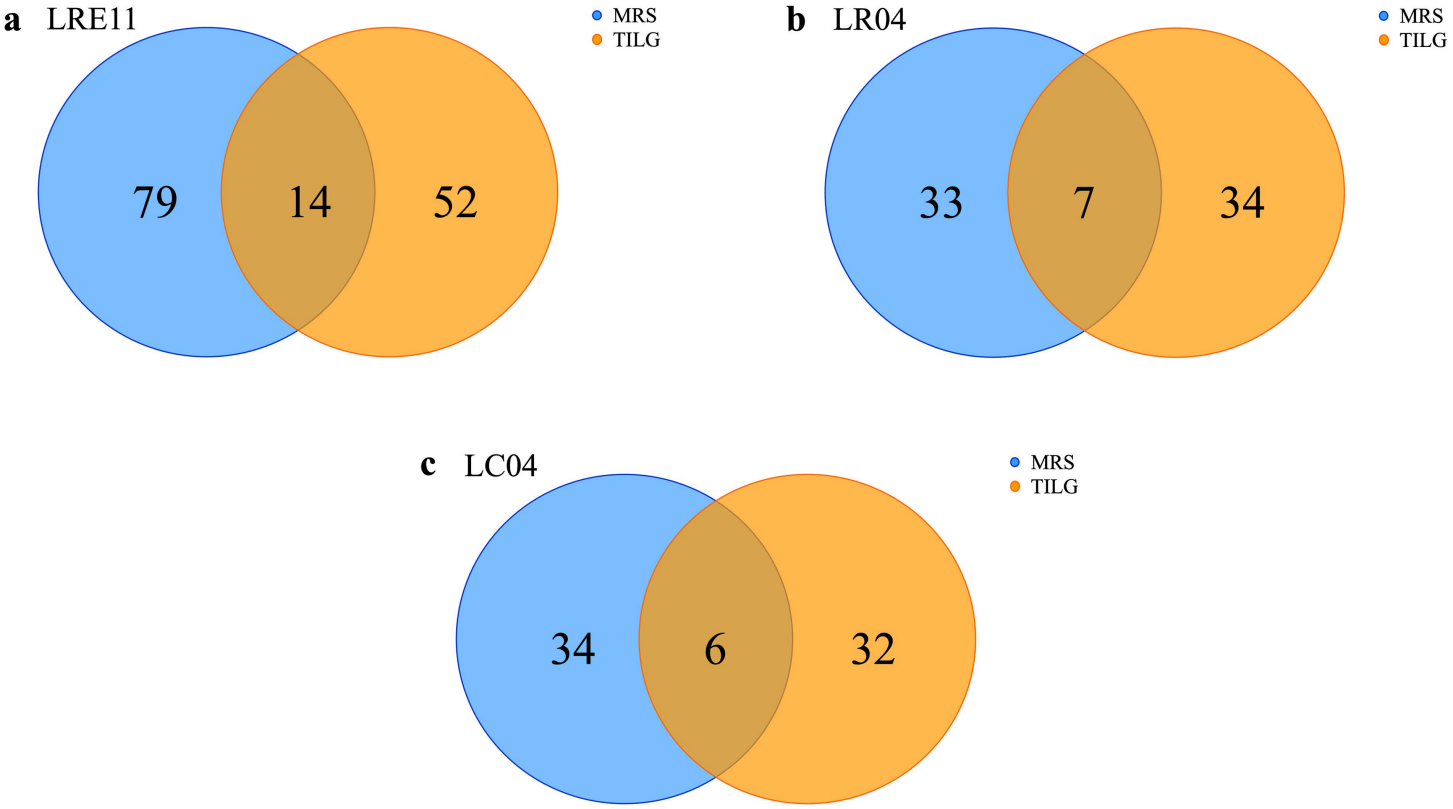
Protein content characterization of *Lactobacillus* CFSs cultivated ON in MRS and TIL media. Identified proteins in MRS and TIL media for **(a)** LRE11, **(b)** LR04, and **(c)** LC04 CFSs.

To identify *Lactobacillus* CFS protein content, produced in both MRS and TIL media after overnight (ON) culture, an untargeted proteomic analysis was performed. LRE11 produced a total of 145 proteins, of which 79 were identified exclusively in the MRS medium, 52 in TILG only, and 14 in both media (Figure 7a). The analysis identified 74 proteins produced by LR04, 33 related to the MRS medium and 34 to TILG, with 7 in common (Figure 7b). For LC04, 34 different proteins were identified in MRS and 32 in TILG, with 6 shared between both media, for a total of 72 proteins identified (Figure 7c). Notably, LR04 and LC04 CFSs exhibited similar protein amounts identified in both media.

**Figure 8:**
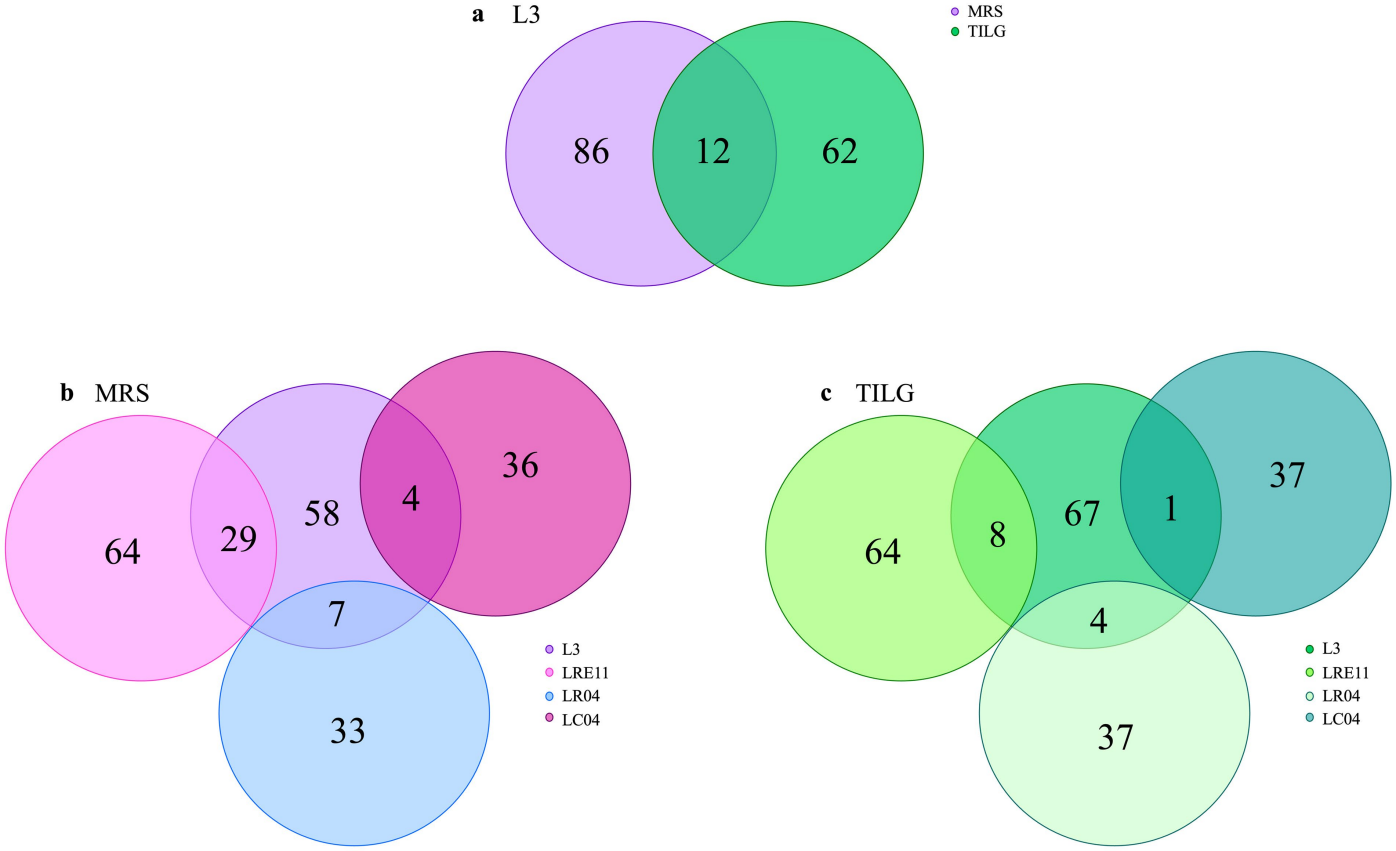
Protein content characterization of L3 CFSs cultivated ON in MRS and TIL media. Proteins identified in MRS and TIL media for **(a)** L3 CFSs. Comparison between the protein content of L3 and of the single probiotic strain CFSs produced in **(b)** MRS and **(c)** TILG.

Similarly, to what was done for each *Lactobacillus* CFS protein identification, the analysis was repeated on L3 CFSs produced in both MRS and TIL media after ON culture. The analysis identified a total of 160 proteins produced by L3, of which 86 only present in the MRS medium and 62 in TILG, while 12 proteins were common to both media (Figure 8a). Comparing the L3 CFS produced in MRS together with the single *Lactobacillus* strain CFSs in the same medium, it was observed that L3 produced 29, 7, and 4 proteins that were also present in LRE11, LR04, and LC04 CFSs, respectively (Figure 8b). In TILG, fewer common proteins were identified: 8, 4, and 1 protein between LRE11, LR04, and LC04, respectively, and the L3 TIL-produced CFS (Figure 8c). Overall, fewer proteins were identified in the TIL-produced L3 CFS compared to the MRS-produced one. Comparing both MRS-and TIL-produced L3 CFSs with the single probiotic strain ones, the number of proteins identified in L3 was more similar to those found in LRE11 CFSs, and almost double the number identified in LR04 and LC04 CFSs.

In Table 2 all the common proteins identified for each strain cultivated in the two different media are listed. As observed, the identified proteins mainly include ribosomal proteins and enzymes involved in carbon metabolism, such as 2,3-bisphosphoglycerate, phosphogluconate dehydrogenase, glyceraldehyde-3-phosphate dehydrogenase, pyruvate kinase, NAD-dependent glyceraldehyde-3-phosphate dehydrogenase, and enolase. In general, in both media and for all the strains, the main proteins found were those involved in carbon source and energy metabolism, together with several proteins related to ribosomal activity and transport proteins, such as peptide ABC transporter permease, ABC transporter substrate-binding protein, and major facilitator transporter protein. In addition, bacterial enzymes and proteins involved in replication were also identified, such as the septum formation initiator and DNA repair protein RecN. Interestingly, proteins and enzymes involved in bacterial defense against bacteriophage infections were detected, as the phage superinfection immunity protein in LC04 CFS in MRS, the phage head morphogenesis protein in LR04 CFS in MRS, the phage portal protein in LRE11 CFS in MRS, and the phage tail protein in LRE11 CFS in TILG. Other molecules found included elongation factors and adhesins. For example, LC04 produced adhesin in MRS, while LRE11 produced two different types of LPXTG-anchored mucus adhesins in both media. Additionally, LC04 was the only strain to produce metallo-beta-lactamase in MRS, while in TILG, LR04 expressed PBP1A family penicillin-binding protein, beta-lactamase, and antibiotic biosynthesis monooxygenase.

**Table 2:** Common proteins identified in the CFSs produced in both MRS and TIL culture media.

Probiotic strain	N. of identified common proteins	Protein ID
LRE11	14	sp|B2G5K8.1|RL31B_LIMRJ 50S ribosomal protein L31 type B
		sp|B2G5T7.1|RL7_LIMRJ 50S ribosomal protein L7/L12
		OYT00024.1 Phosphogluconate dehydrogenase
		AKP00622.1 Enolase
		AKP00619.1 Glyceraldehyde-3-phosphate dehydrogenase
		OYT00957.1 Hypothetical protein CBG24_03120
		AKP00779.1 Thioredoxin
		AKP00993.1 Pyruvate kinase
		sp|B2G6Q8.1|RS20_LIMRJ 30S ribosomal protein S20
		CCC02982.1 Conjugated bile salt hydrolase
		QWS03614.1 Glycoside hydrolase family 73 protein
		OYT01734.1 Cytochrome B5
		WP_191990789.1 Ubiquitin family protein
		sp|B2G5T1.1|RL11_LIMRJ 50S ribosomal protein L11
LR04	7	EKS50007.1 Glycosyltransferase
		RDJ92975.1 Hypothetical protein B4Q13_25105
		OAU37774.1 Adhesin
		OAU79553.1 Transposase
		WP_188434125.1 5-(carboxyamino)imidazoleribonucleotidesynthase
		UTX29435.1 Type I glyceraldehyde-3-phosphate dehydrogenase
		OAU73993.1 hypothetical protein PY62_14940
LC04	6	OLS10371.1 FliK family flagellar hook-length control protein
		OLS10644.1 Hypothetical protein AUQ39_03195
		QXG59908.1 KxYKxGKxW signal peptide domain-containing protein
		OLS10606.1 Hydrolase
		EKQ00297.1 NAD-dependent glyceraldehyde-3-phosphate dehydrogenase
		EKQ03636.1 CspA family cold shock protein

In Table 3 all the common proteins identified in the L3 CFS produced in MRS and TILG media are listed. They are mainly ribosomal proteins and enzymes involved in carbon source metabolism, such as glyceraldehyde-3-phosphate dehydrogenase, enolase, and hydrolase.

**Table 3:** Common proteins identified in the L3 CFSs produced in both MRS and TIL culture media.

Probiotic strain comparison	N. of identified common proteins	Protein ID
L3 MRS vs. L3 TILG	12	sp|B2G856.1|RL27_LIMRJ 50S ribosomal protein L27
		sp|B2G5T7.1|RL7_LIMRJ 50S ribosomal protein L7/L12
		AKP00622.1 Enolase
		sp|B2G8X3.1|RL22_LIMRJ 50S ribosomal protein L22
		AXX74097.1 DUF1542 domain-containing protein
		AKP00779.1 Thioredoxin
		AEI57815.1 Glyceraldehyde-3-phosphate dehydrogenase, type I
		ONG00067.1 Type I glyceraldehyde-3-phosphate dehydrogenase
		WP_191990789.1 ubiquitin family protein
		AKP00649.1 Peptidoglycan-binding LysM
		OLS10606.1 Hydrolase
		WP_229394423.1 MucBP domain-containing protein

Table 4 reports the proteins found in both L3 CFS and that of each probiotic strain in the same MRS medium. Similarly, ribosomal proteins are the most prevalent, followed by enzymes related to carbon metabolism.

**Table 4:** Common proteins identified in L3 and LRE11, LR04, and LC04 CFSs produced in MRS medium.

Probiotic strain comparison	N. of identified common proteins	Protein ID
L3 MRS vs. LRE11 MRS	29	AWD62211.1 Argininosuccinate lyase
		WP_285228057.1 Hypothetical protein
		sp|B2G8W7.1|RL24_LIMRJ 50S ribosomal protein L24
		AKP00807.1 Hypothetical protein LRIRT_0582
		sp|B2G8X4.1|RS19_LIMRJ 30S ribosomal protein S19
		sp|B2G5X6.1|CH10_LIMRJ Chaperonin-10
		UYQ75981.1 Lar_0958 family LPXTG-anchored mucus adhesin
		sp|B2G6R3.1|TIG_LIMRJ PPIase
		QQR14360.1 BspA family leucine-rich repeat surface protein
		AGO00140.1 Pyruvate/2-oxoglutarate dehydrogenasecomplex, dihydrolipoamidedehydrogenase (E3) component (plasmid)
		AKP01373.1 Acyl carrier protein
		sp|B2G649.1|G6PI_LIMRJ Phosphohexose isomerase
		sp|B2G8W6.1|RL5_LIMRJ 50S ribosomal protein L5
		OTA48908.1 Dextransucrase
		ROV63130.1 Hypothetical protein EGO58_05570
		OYT01536.1 Nucleoside hydrolase
		OYT01540.1 DNA starvation/stationary phase protection protein
		AKP00866.1 NLP/P60 protein
		AKP00731.1 Glyoxalase/bleomycin resistance protein/dioxygenase
		PWT39145.1 Phage portal protein
		AKP01716.1 Cold-shock DNA-binding protein family protein
		sp|B2G8X6.1|RL23_LIMRJ 50S ribosomal protein L23
		sp|A5VLI8.1|RS5_LIMRD 30S ribosomal protein S5
		OYT00187.1 ISL3 family transposase
		sp|B2G4Z1.1|RS6_LIMRJ 30S ribosomal protein S6
		OYT00955.1 Hypothetical protein CBG24_03110
		OTA78368.1 Serine protease
		UCN18289.1 Glycosyltransferase
		CCC02995.1 L-lactate dehydrogenase
L3 MRS vs. LR04 MRS	7	OAU07811.1 Transposase
		UTX30164.1 UDP-N-acetylglucosamine 1-carboxyvinyltransferase
		WP_154244675.1 FtsX-like permease family protein
		ASX17610.1 Hypothetical protein BGK71_09380
		EGF48212.1 Lipoprotein (pheromone precursor)
		OAU00496.1 Major facilitator transporter
		WP_176818387.1 head-tail connector protein
L3 MRS vs. LC04 MRS	4	QVI36164.1 Cell wall hydrolase P75
		OLS04121.1 Hypothetical protein AUQ39_14320
		QVI36410.1 cell wall hydrolase P40
		EKQ10985.1 Phage integrase

Table 5 outlines the shared proteins from the CFSs produced in TILG, where, notably, no common ribosomal proteins were identified. Instead, various enzymes such as transposase, endonuclease, and kinases were present. In general, in both media the main proteins identified were the ones involved in carbon sources and energetic metabolism, together with several proteins related to ribosomal activity and transporter proteins, such as ABC transporter ATP-binding protein and major facilitator transporter protein, as for the single strains previously discussed. In this case, bacterial enzymes and proteins involved in replication were not present in the L3 CFS, while proteins and enzymes involved in bacterial defense against bacteriophage infections, like phage portal and tail proteins, were still detected. No elongation factors were identified, but LPXTG-anchored mucus adhesin was found in both MRS-and TILG-produced L3 CFSs, with LEA family epithelial adhesion protein exclusively retrieved in TILG. Interestingly, none of the bacteria in the L3 blend produced proteins or enzymes associated with antibiotic resistance in either medium. However, when the three probiotic strains (LRE11, LR04, and LC04) were co-cultivated to form the L3 blend, LRE11 synthesized the muramidase enzyme in MRS, which it only produced as a single strain when grown in TILG.

**Table 5:** Common proteins identified in both L3 and LRE11, LR04, and LC04 CFSs produced in TILG medium.

Probiotic strain comparison	N. of identified common proteins	Protein ID
L3 TILG vs. LRE11 TILG	8	WP_229410725.1 DUF4355 domain-containing protein
		EGC14139.1 Polysaccharide biosynthesis protein
		WP_231127639.1 Lar_0958 family LPXTG-anchored mucus adhesin
		WP_229279544.1 IS30 family transposase
		UNL36914.1 HaeIII family restriction endonuclease
		WP_229270455.1 Hypothetical protein
		PWT67750.1 Thymidine kinase
		WP_267495658.1 MDR family MFS transporter
L3 TILG vs. LR04 TILG	4	WP_233026229.1 Hypothetical protein
		RDJ93191.1 Hypothetical protein B4Q13_23595
		RDJ93070.1 Hypothetical protein B4Q13_24490
		PTS00478.1 Hypothetical protein DBQ08_14490
L3 TILG vs. LC04 TILG	1	EKQ01193.1 Alpha/beta superfamily hydrolase

### 2.8 Probiotic CFS analysis of the SCFA content

**Figure 9:**
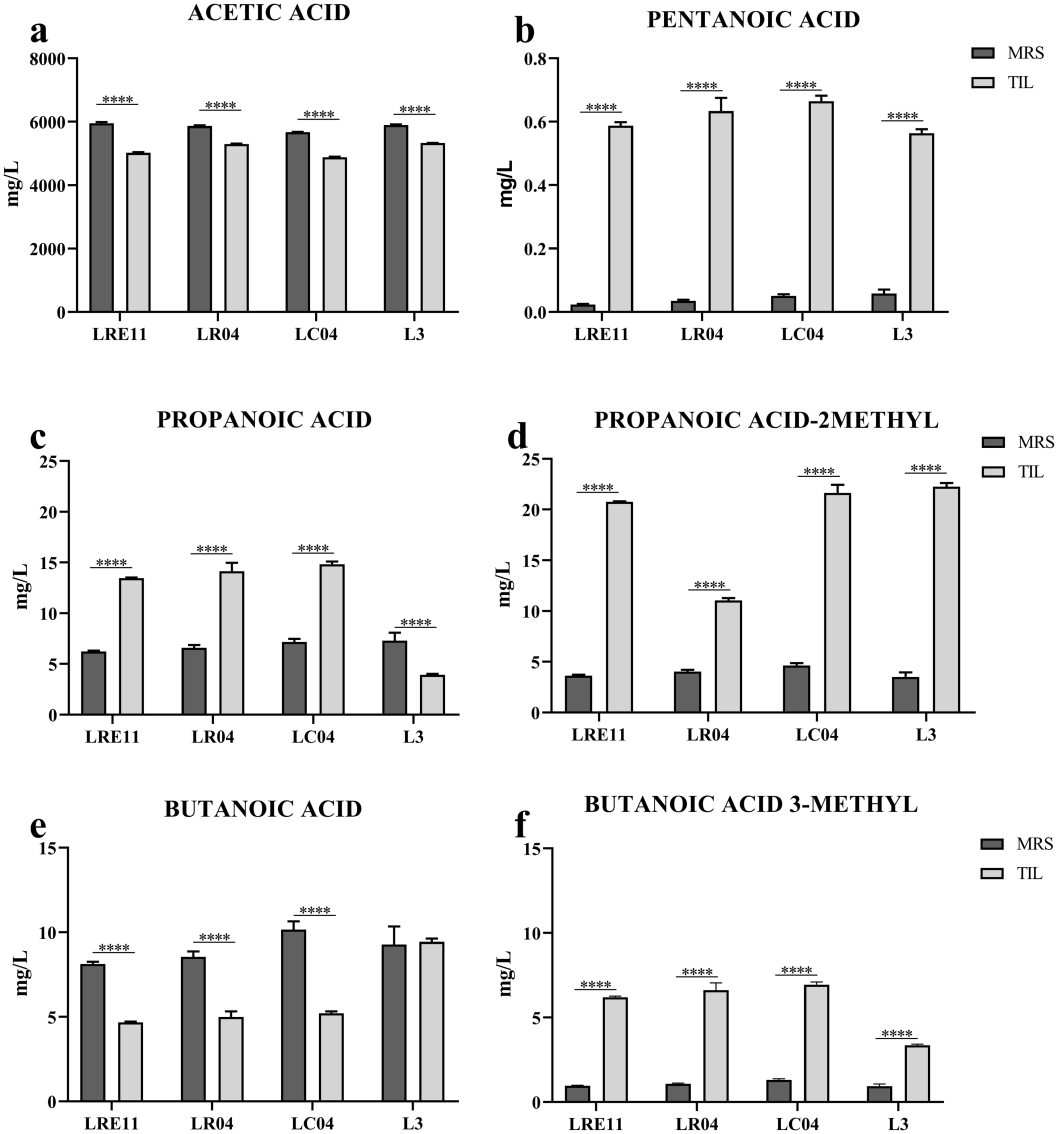
SCFA content characterization of LRE11, LR04, LC04, and L3 CFSs cultivated ON in MRS and TIL media. Graphs represent the mean ± SD of **(a)** acetic acid, **(b)** pentanoic acid, **(c)** propanoic acid, **(d)** propanoic acid 2-methyl, **(e)** butanoic acid, and **(f)** butanoic acid 3-methyl evaluated three times independently for each sample. ****p < 0.0001.

In Figure 9, the SCFA content determined for the probiotic CFSs are shown. As for lactic acid and protein production, the different growth conditions resulted in specific variations in SCFA production.

Specifically, acetic acid production was significantly lower in all probiotics and the L3 grown in TIL when compared to MRS, as seen in Figure 9a. LC04 CFSs produced in both TIL and MRS showed the lowest acetic acid production when compared to the other strains and the L3 (*p* < 0.0001; Figure 9a).

On the other side, pentanoic acid production significantly increased when all probiotics and L3 were cultivated in TIL instead of MRS (Figure 9b). No differences were observed in its concentration in MRS, while in TIL, LRE11 and L3 CFSs produced the lowest pentanoic acid levels (*p* < 0.05 and *p* < 0.001 vs. LR04 and LC04 for LRE11; *p* < 0.01 and *p* < 0.0001 for L3; Figure 9b).

Propanoic acid levels significantly increased in TIL for all probiotics except for L3, which showed a higher concentration when produced in MRS (Figure 9c). The propanoic acid content was similar among all MRS-produced CFSs, with L3 CFS showing a slightly higher concentration compared to the one of LRE11 (*p* < 0.05, Figure 9c). In TIL, instead, the L3 CFS showed the lowest concentration (*p* < 0.0001 vs. all other TIL-CFSs; Figure 9c).

Propanoic acid 2-methyl production was higher when the probiotics and L3 were grown in TIL (Figure 9d). While production was almost equal across all CFSs in MRS, LR04 CFS had the lowest concentration in TIL (*p* < 0.0001 vs. all other TIL-CFSs; Figure 9d).

Butanoic acid levels remained unchanged for L3 CFS regardless of the different media used, but decreased significantly for single strains when cultivated in TIL (Figure 9e). L3 CFS produced in TIL had the highest butanoic acid concentration compared to single strains (*p* < 0.0001; Figure 9e).

Butanoic acid 3-methyl production was significantly higher for all CFSs grown in TIL (Figure 9f), with LC04 CFS having the highest concentration (*p* < 0.0001) and L3 CFS the lowest (*p* < 0.0001) in TIL.

## 3 Discussion

Considering the pivotal role of the oral microbiota in maintaining oral health and improving disease outcomes, strategies aimed at preserving or restoring oral microbiota eubiosis can enhance the efficacy of pharmacological treatments and overall oral health management (Zaura et al., 2014; Sivan et al., 2015). In this study, we explored the beneficial effects of cell-free supernatants (CFSs) from the probiotics *L. reuteri* LRE11, *L. rhamnosus* LR04, and *L. casei* LC04, and their co-culture (L3) on a human hypopharyngeal squamous carcinoma cell line (FaDu), infected with the oral pathogens *A. actinomycetemcomitans*, *F. nucleatum*, and *P. gingivalis*.

FaDu cells are widely used for *in vitro* studies to assess inflammatory responses in both infected and non-infected contexts of both the oral niche and pharynx (Brú et al., 2009; Persson et al., 2024). Given that these oral pathogens infect both the oral mucosa and the pharynx, and that FaDu cells express key receptors like IL-6, they are suitable for evaluating the effect of probiotic CFSs and vitamin D during infection extending beyond the oral cavity, ensuring reproducible results (Chen et al., 2010).

These pathogens are known contributors to oral disease onset and tumorigenesis (Kamarajan et al., 2020). Recognizing the crucial role of vitamin D in supporting a health gut microbiota (Pludowski et al., 2013), we also investigated its potential synergistic effect with probiotics.

Probiotics were cultured in two different media, MRS and TIL, to assess how growth conditions may influence bacterial metabolism and metabolites production (Raveschot et al., 2020; Papadimitriou et al., 2015). An initial 4 h-viability assay with probiotic CFSs and vitamin D was conducted to identify non-toxic CFS concentrations on FaDu cells. Based on these results, the concentrations of 20, 10, and 5% v/v were selected for further experiments, ensuring that observed outcomes could be attributed to the probiotic metabolites rather than any cytotoxic effects.

On the viability assay on infected FaDu cells, the probiotic CFSs significantly reduced pathogen-induced cytotoxicity. Notably, the MRS-produced CFSs demonstrated superior protective activity compared to those produced in TIL, with exceptions for LR04 and LC04. The co-culture of the three probiotic strains (L3) yielded the most remarkable results, especially at the 20% concentration of the MRS-produced L3 CFS, which effectively neutralized the cytotoxicity caused by co-infection with the three pathogens.

These findings align with previous studies where probiotic combinations showed enhanced efficacy in inhibiting pathogenic bacteria and reducing cytotoxic effects (Pahumunto et al., 2020).

Our goal was to closely simulate a realistic polymicrobial infection, where these three pathogens interact synergistically, influencing each other’s metabolism, growth and biofilm formation, reflecting the natural complexity of infections. Additionally, our co-infection model enhances IL-6 production, further amplifying the effects induced by the probiotic mix and vitamin D.

In this direction, when probiotic CFSs were applied to non-infected FaDu cells, they reduced basal IL-6 levels, indicating their inherent anti-inflammatory properties. This underscores the potential of probiotics to modulate inflammatory responses even in tumoral cells. Similar anti-inflammatory effects have been reported in other studies (De Marco et al., 2018; Hao et al., 2023) both on human and mouse cell *in vitro* models. For instance, *L. rhamnosus* GG was shown to reduce IL-6 levels in intestinal and liver epithelial cells, contributing to amelioration of inflammation (Nenu et al., 2024).

In infected FaDu cells, the combination of probiotic CFSs and vitamin D significantly enhanced the anti-inflammatory response. Some treatments not only mitigated the infection-induced rise of the early inflammatory marker IL-6, which is crucial to restore eubiosis and modulate immunity, but also reduced its levels below those of untreated cells.

This indicates a potent synergistic anti-inflammatory effect between probiotics and vitamin D. Recent findings suggest that vitamin D can enhance the immunomodulatory effects of probiotics, further supporting our observations (Kang et al., 2011).

Recognizing the potential for combined probiotic supplementation to exert broader effects on oral microbiota and the tumoral microenvironment, we further investigated the active components within the CFSs responsible for these beneficial effects. Understanding these components could pave the way for developing targeted probiotic therapies for oral health. To this end, the L3 CFSs were digested with proteinase K and trypsin, and the viability and ELISA experiments were repeated. The digested L3 CFSs maintained their protective effects, suggesting that both protein and non-protein components contribute to reducing pathogen cytotoxicity and inflammation.

The significant reduction in IL-6 production may be attributed to the production of short-chain fatty acids (SCFAs) by the probiotic strains, known for their anti-inflammatory properties (Feitelson et al., 2023). SCFAs, such as butyric acid and propionic acids, play crucial roles in maintaining mucosal immunity and have been recognized for their capacity to modulate inflammatory responses (McNabney and Henagan, 2017). The observed reduction in IL-6 levels, even after enzymatic digestion of the CFSs, suggests that SCFAs or other non-proteinaceous metabolites may be key mediators of the probiotic anti-inflammatory action. This highlights the potential of harnessing these metabolites for the therapeutic purposes.

These *in vitro* findings have significant clinical implications. The demonstrated ability of these probiotics, especially when combined, to reduce pathogen-induced cytotoxicity and inflammation in FaDu cells suggests that they could be developed into effective probiotic-based interventions for oral diseases. Such interventions could complement existing therapies, enhance treatment efficacy, and potentially reduce reliance on antibiotics, thereby mitigating concerns related to antibiotic resistance. Probiotic therapies have gained attention in oral health management, with studies showing their benefits in managing periodontal diseases and reducing pathogen load (Onyszkiewicz et al., 2020; Shen et al., 2024; Li et al., 2024; Kim et al., 2020). Our findings extend this potential to include protective effects against oral pathogens associated with tumorigenesis.

Nevertheless, despite FaDu cells are an optimal cell model, they do not recapitulate the complexity of the *in vivo* oral niche where also other cells, such as fibroblasts, immune cells, and the extracellular matrix, together with the microbial competition and the host’s response, different from one individual to another, have an important role.

Future research should also focus on identifying and quantifying the specific SCFAs and other active metabolites responsible for these beneficial effects. Determining the most effective types and concentrations of SCFAs could inform the development of targeted probiotic formulations or supplements. Additionally, investigating the mechanisms by which these metabolites exert their effects could provide deeper insights into their potential therapeutic applications. Exploring the use of viable and heat-killed probiotic strains in comparison to CFSs will also be crucial to fully understand their interactions with host cells and the resulting impact on oral health.

Furthermore, deeper *in vitro* investigations will be essential to elucidate how probiotics synergically interact with vitamin D. Moreover, further clinical *in vivo* research will be essential to validate these findings to assess their use for the management of endogenous and exogenous oral infections.

In conclusion, our study demonstrates that probiotics, particularly when combined and supplemented with vitamin D, can significantly reduce pathogen-induced cytotoxicity and inflammation in FaDu epithelial cells. These positive outcomes not only contribute to the growing body of evidence supporting the use of probiotics in oral health, but also open new avenues for developing innovative therapeutic strategies. By enhancing our understanding of the active components and mechanisms involved, we can move closer to implementing effective probiotic-based interventions that improve oral health and potentially influence the tumor microenvironment favorably.

## 4 Materials and methods

### 4.1 Eukaryotic cell culture

A human hypopharyngeal squamous carcinoma cell line (FaDu; HTB-43, American Type Culture Collection, ATCC 43300, distributed by LGC Standards S.r.l., Sesto San Giovanni, Milan, Italy) was cultivated in Dulbecco’s Modified Eagle’s Medium (DMEM; Cytiva, Logan, Utah, United States, distributed by CliniSciences S.r.l., Guidonia Montecelio, Rome, Italy) with L-glutamine (4 mM), and high glucose concentration (4,500 mg/L), without sodium pyruvate, and supplemented with 10% heat-inactivated fetal bovine serum (FBS; Corning, Glendale, Arizona, USA, distributed by Biosigma S.p.A., Cona, Venice, Italy), and 1% penicillin and streptomycin mixture (10,000 units/mL penicillin and 10 mg/mL streptomycin mixture, Sigma-Aldrich, St. Louis, MO, USA, distributed by Merck Life Science S.r.l., Milan, Italy). The cells were kept in a humidified 5% CO_2_ atmosphere at 37°C. For all the experiments, FaDu cells were seeded into 96-well plates (1 × 10^5^ cells/mL, 100 μL/well), in complete growth medium without antibiotics. Cells were PCR-tested to exclude mycoplasma contamination every 4 weeks.

### 4.2 Bacterial cultures

*Aggregatibacter actinomycetemcomitans* (DSM 11123, Deutsche Sammlung von Mikroorganismen und Zellkulturen, DSMZ, Braunschweig, Germany) was aerobically cultivated overnight (ON) at 37°C and 200 rpm in tryptic soy broth (TSB, Sigma-Aldrich). *Fusobacterium nucleatum* (DSM 15643) and *Porphyromonas gingivalis* (DSM 20709) were grown at 37°C in anaerobic 2.5 L rectangular jars with Oxoid™ AnaeroGen™ sachets (Thermo Fisher Diagnostic S.p.A.) ON using brain hearth infusion broth (BHI, Sigma-Aldrich) supplemented with 0.5% N-acetyl-L-cysteine (Sigma-Aldrich), 5 μg/mL hemin (Sigma-Aldrich), and 0.5 μg/mL menadione (Sigma-Aldrich). FaDu cells were infected with 100 multiplicities of infection (MOI) of a blend of the above pathogens. Briefly, each strain was freshly renewed the day before each experiment, then the culture was centrifuged at 4,000 rpm, room temperature (RT), for 15 min and the pellet was washed with PBS 1X to completely remove the prokaryotic medium. After another centrifugation step, the pellet was resuspended in DMEM, without FBS and antibiotics, at 10^9^ CFU/mL, and the three pathogens were then co-diluted.

The probiotic strains *Limosilactobacillus reuteri* LRE11 (DSM 33827), *Lacticaseibacillus rhamnosus* LR04 (DSM 16605), and *Lacticaseibacillus casei* LC04 (DSM 33400), kindly provided by Probiotical Research S.r.l., Novara, Italy, were aerobically grown in static conditions ON at 37°C, using the standard animal derivative-based De Man, Rogosa and Sharpe broth (MRS, Condalab, distributed by Cabru S.A.S., Biassono, Italy) and the animal derivative-free medium, generically referred as “Terreno Industriale Lattobacilli” (TIL) broth (Probiotical Research S.r.l.; formula in g/L: proteose peptone N-3 10, dextrose 20, dipotassium phosphate 2, magnesium sulfate 0.1, manganese sulfate 0.05, vegetal extract-confidential, sodium acetate 5, Tween-80 1, yeast extract 5, ammonium citrate 2), containing peptones from plant sources, supplemented with glucose (TILG).

### 4.3 Cell-free supernatant (CFS) production

LRE11, LR04, and LC04 fresh cultures were inoculated at an optical density at 600 nm (OD_600_) = 0.05 into MRS or TILG broth and incubated ON in proper conditions. Then, probiotic growth was assessed through OD_600_ measurement, and the bacterial culture was centrifuged at 4,000 rpm for 20 min at 4°C. To prevent the acidity confounding effect, the CFSs pH was neutralized to a pH value of 7 with 5 N NaOH (Sigma-Aldrich), before sterilizing them with 0.22 μm polyethersulfone (PES) filters (Clearline, distributed by Biosigma), aliquoted, and stored at −20°C until use. Considering that, in real conditions, people are often supplemented with more than one probiotic species at a time, a three-strain co-culture was developed (L3). Briefly, the single probiotic strains were mixed in MRS or TILG media and let them adapt and grow through at least three passages in culture. Then, a CFS from this blend was prepared as described above. To obtain information about the protein activity within the L3 CFSs, they were treated with the proteolytic trypsin (TRY, Corning, distributed by Biosigma) or proteinase K (PK) enzymes (Euroclone S.p.A., Pero, Milan, Italy). Briefly, each CFS pH was adjusted at 8 using NaOH 5 N, then it was incubated for 90 min at 37°C either with TRY or PK at the final concentration of 50 μg/mL. After that, the pH was brought to 7 with HCl to maintain the same testing conditions as for the non-digested L3 CFSs. All the CFSs were then filtered with 10 kDa cut-off spin columns (Amicon Ultra-0.5 Centrifugal Filter Unit, Millipore, distributed by Sigma Aldrich) to remove the enzymes (molecular weights in kDa: TRY 24, and PK 28.9, respectively). LRE11, LR04, LC04, and L3 CFS protein content was quantified with the bicinchoninic acid (BCA) Protein Assay Kit (Biosciences, St. Louis, USA, distributed by Cabru S.A.S.), while the lactic acid concentration with the D/L-Lactic Acid Megazyme Assay Kit (NEOGEN Europe Ltd., Ayr, UK), following the manufacturer’s instructions. Pristine MRS and TILG media were incubated, centrifuged, filtered, and stored as the CFS, and used as controls in the following experiments (iMRS and iTILG, respectively).

### 4.4 Viability assay

The CellTiter-Glo® Luminescent Cell Viability Assay (Promega, Italia S.r.l., Milan, Italy) was performed at 4 h following the manufacturer’s instructions on FaDu cells treated with different probiotic CFS v/v ratios (50, 40, 30, 20, 10, and 5%), together with iMRS and iTILG, to select non-toxic conditions for this cell line. Briefly, the day of the experiment the cell medium was changed with fresh DMEM without FBS and antibiotics, and probiotic CFSs were added (final volume/well = 200 μL). Subsequently, a viability assay was also made on FaDu cells infected with the pathogen blend at 100 MOI, and simultaneously treated with the probiotic CFSs (20, 10, and 5%) and/or vitamin D 100 nM in dimethyl sulfoxide (DMSO; Cabru S.A.S) for 4 h in aerobiosis to assess whether the treatments could affect cell viability upon infection. Each experiment was performed in triplicate and repeated three times independently.

### 4.5 IL-6 ELISA assay

For the quantitative detection of IL-6 released from FaDu cells after the infection with the pathogen mixture and/or probiotic CFSs and/or with vitamin D treatment, a commercial human IL-6 ELISA Kit (FineTest ®, Wuhan, China; distributed by Cabru S.A.S.) was used. Briefly, FaDu cells were seeded into 96-well plates and infected and/or treated as described above and incubated for 4 h. Then, the supernatants were collected and centrifuged for 5 min at 2,500 rpm at 4°C to remove insoluble impurities and cell debris and frozen at −80°C until IL-6 quantification following the manufacturer’s instructions. Water-dissolved lipopolysaccharide (LPS; Cabru S.A.S.) at 10 ng/mL was used as inflammation inducer control.

### 4.6 *Lactobacillus* CFS proteomic characterization and short-chain fatty acid analysis

#### 4.6.1 Sample preparation

The CFS proteins were precipitated ON at −20°C with 4 volumes of ice-cold acetone. The pellets were then collected by centrifugation at 17,000 × *g* for 20 min at 4°C and then resuspended in 100 mM ammonium bicarbonate (NH_4_HCO_3_). Protein concentration was determined using the BCA protein assay (Sigma-Aldrich). Proteins were reduced with DTT 200 mM, subjected to alkylation with iodoacetamide (IAM) 200 mM, and then completely digested with 2 μg of TRY. The peptide digests were desalted on the Discovery® DSC-18 solid-phase extraction (SPE) 96-well plate (25 mg/well; Sigma-Aldrich) (Albanese et al., 2018).

#### 4.6.2 Proteomic analysis and data processing

Digested peptides were dried by Speed Vacuum, desalted, and analyzed on an Ultimate 3,000 RSLC nano coupled directly to an Orbitrap Exploris 480 with a High-Field Asymmetric Waveform Ion Mobility Spectrometry System (FAIMSpro) (all Thermo Fisher Scientific). Samples were injected onto a reversed-phase C18 column (15 cm × 75 μm i.d., Thermo Fisher Scientific) and eluted with a gradient of 6–95% mobile phase B over 80 min by applying a flow rate of 300 nL/min, followed by an equilibration with 6% mobile phase B for 8 min. Mass spectrometry (MS) scans were performed in the range of *m/z* 375–1,200 at a resolution of 120.000 (at *m/z* = 200). MS/MS scans were performed choosing a resolution of 15.000; normalized collision energy of 30%; isolation window of 2 *m/z*; and dynamic exclusion of 45 ns. Two different FAIMS compensation voltages were applied (−45 V and −60 V), with a cycle time of 1.5 s per voltage. FAIMS was operated in standard resolution mode with a static carrier gas flow of 4.6 L/min. The acquired raw MS data files were processed and analyzed using Proteome Discoverer (v3.0.0.757, Thermo Fisher Scientific). SequestHT was used as a search engine and the following parameters were chosen. Enzyme: TRY; max. missed cleavage sites: 2; static modifications: carbamidomethyl (C); dynamic modifications: oxidation (M); precursor mass tolerance: 10 ppm; fragment mass tolerance: 0.02 Da. Only peptides and proteins with FDR value < 0.01 were reported. Database: *Limosilactobacillus reuteri* LRE11 (DSM 33827); *Lacticaseibacillus rhamnosus* LR04 (DSM 16605); *Lacticaseibacillus casei* LC04 (DSM 33400), downloaded from NCBI on 06.06.2023.

#### 4.6.3 Short-chain fatty acid analysis

Basal culture media and CFSs were assessed for SCFAs content after a liquid–liquid extraction method with methyl tert-butyl ether (MTBE). SCFAs were then analyzed using a gas chromatography-mass spectrometer GC-TOFMS (BT, Leco Corp., St. Josef, MI, USA), as previously described (Barberis et al., 2021). Briefly, the column adopted was a 30 m DB-FATWAX-UI (Agilent Technologies, Santa Clara, CA, USA), while high-purity helium (99.9999%) was used as the carrier gas. One μL of each sample was injected in splitless mode at 250°C. The program was as follows: the initial temperature was 40°C for 2 min, then ramped 7°C/min up to 165°C, 25°C/min up to 240°C, and maintained for 5 min. The electron impact ionization was applied at 70 eV. The ion source temperature was set at 250°C, the mass range at 40–300 *m/z* with an extraction frequency of 32 kHz and an acquisition rate of 200 spectra/s.

## 5 Statistical analysis

One-way and two-way ANOVA, followed by Tukey multiple comparisons, were performed using the GraphPad Prism version 7.04 for Windows (GraphPad Software, San Diego, California USA).^1^ Results were expressed as mean ± standard deviation (SD). Statistical significance was fixed at *p* < 0.05.

## Data Availability

The original contributions presented in the study are publicly available. This data can be found here: https://www.proteomexchange.org/, PXD059098.

## Footnotes

^1^ www.graphpad.com
